# A Systematic Review of the Spectrum of Cardiac Arrhythmias in Sub-Saharan Africa

**DOI:** 10.5334/gh.808

**Published:** 2020-05-08

**Authors:** Matthew F. Yuyun, Aimé Bonny, G. André Ng, Karen Sliwa, Andre Pascal Kengne, Ashley Chin, Ana Olga Mocumbi, Marcus Ngantcha, Olujimi A. Ajijola, Gene Bukhman

**Affiliations:** 1 Department of Medicine, Harvard Medical School, Boston, US; 2 Cardiology and Vascular Medicine Service, VA Boston Healthcare System, Boston, US; 3 District Hospital Bonassama, Douala/University of Douala, CM; 4 Homeland Heart Centre, Douala, CM; 5 Centre Hospitalier Montfermeil, Unité de Rythmologie, Montfermeil, FR; 6 National Institute for Health Research Leicester Biomedical Research Centre, Department of Cardiovascular Sciences, University of Leicester, UK; 7 Hatter Institute for Cardiovascular Research in Africa, University of Cape Town, ZA; 8 South African Medical Research Council and Department of Medicine, University of Cape Town, ZA; 9 The Cardiac Clinic, Department of Medicine, Groote Schuur Hospital and University of Cape Town, ZA; 10 Instituto Nacional de Saúde and Universidade Eduardo Mondlane, Maputo, MZ; 11 Ronald Reagan UCLA Medical Center Los Angeles, US; 12 Division of Cardiovascular Medicine and Division of Global Health Equity, Brigham and Women’s Hospital, Boston, US; 13 Program in Global NCDs and Social Change, Department of Global Health and Social Medicine, Harvard Medical School, Boston, US; 14 NCD Synergies project, Partners In Health, Boston, US

**Keywords:** atrial arrhythmias, supraventricular tachycardia, sudden cardiac death, ventricular arrhythmias, pacemaker, defibrillator, sub-Saharan Africa

## Abstract

Major structural cardiovascular diseases are associated with cardiac arrhythmias, but their full spectrum remains unknown in sub-Saharan Africa (SSA), which we addressed in this systematic review. Atrial fibrillation/atrial flutter (AF/AFL) prevalence is 16–22% in heart failure, 10–28% in rheumatic heart disease, 3–7% in cardiology admissions, but <1% in the general population. Use of oral anticoagulation is heterogenous (9–79%) across SSA. The epidemiology of sudden cardiac arrest/death is less characterized in SSA. Cardiopulmonary resuscitation is challenging, owing to low awareness and lack of equipment for life-support. About 18% of SSA countries have no cardiac implantable electronic devices services, leaving hundreds of millions of people without any access to treatment for advanced bradyarrhythmias, and implant rates are more than 200-fold lower than in the western world. Management of tachyarrhythmias is largely non-invasive (about 80% AF/AFL via rate-controlled strategy only), as electrophysiological study and catheter ablation centers are almost non-existent in most countries.

**Highlights:**

- Atrial fibrillation/flutter prevalence is 16–22% in heart failure, 10–28% in rheumatic heart disease, 3–7% in cardiology admissions, and <1% in the general population in sub-Saharan Africa (SSA).
- Rates of oral anticoagulation use for CHA2DS2VASC score ≥2 are very diverse (9–79%) across SSA countries.
- Data on sudden cardiac arrest are scant in SSA with low cardiopulmonary resuscitation awareness.
- Low rates of cardiac implantable electronic devices insertions and rarity of invasive arrhythmia treatment centers are seen in SSA, relative to the high-income countries.

## Introduction

About 1.1 billion people live in 49 countries of sub-Saharan Africa (SSA) accounting for approximately 15% of the world population [1]. Historically, the leading causes of mortality in SSA have been communicable diseases, and non-communicable diseases (NCDs) were not considered to be a public health priority [2,3]. However, it is now clear that the burden of NCDs has long been an endemic problem in SSA [3,4,5,6]. NCDs are projected to overtake infectious diseases and account for more than half of all deaths by 2030 in SSA [7]. Among NCDs, cardiovascular diseases (CVDs) are the leading cause of death accounting for 37% of all NCDs deaths and approximately 13% of all deaths in SSA [3]. Approximately 32 million people in SSA are currently living with some form of diagnosed CVD and it is estimated that there are many more living with undiagnosed CVD, and 3.6 million incident cases are reported annually [2,3].

All major structural CVDs are associated with cardiac arrhythmia. Therefore, it is likely that the growing burden of CVDs in SSA also reflects the growing burden of cardiac arrhythmias, though the full spectrum is poorly characterized. Prior reviews have described the epidemiology of atrial fibrillation in Africa [8]. However, it remains uncertain as to whether atrial fibrillation/atrial flutter (AF/AFL), supraventricular tachycardias (SVTs), significant bradyarrhythmias, ventricular tachycardia/ventricular fibrillation (VT/VF), as well as sudden cardiac arrest/sudden cardiac death (SCA/SCD) are regularly diagnosed and treated in SSA. Ascription of SCD among some SSA populations to non-medical causes like witchcraft is not uncommon [9]. Nonetheless, arrhythmias are probably underdiagnosed in SSA due to lack of equipment and expertise [10]. Few informative surveys and reviews on the status of arrhythmia services in Africa have revealed severe deficiencies in healthcare systems and arrhythmia specialists [9,11,12,13,14]. However, a detailed description of clinical arrythmia entities in SSA is lacking. This systematic review will therefore assess the distribution, etiologies, diagnosis, and treatment of arrhythmias in tandem arrythmia services in SSA. It will also compare these with high-income countries (HIC) of Western Europe and North America, identify possible contributors to any under-diagnosis and under-treatment, and provide some recommendations.

## Methods

**Figure 1:**
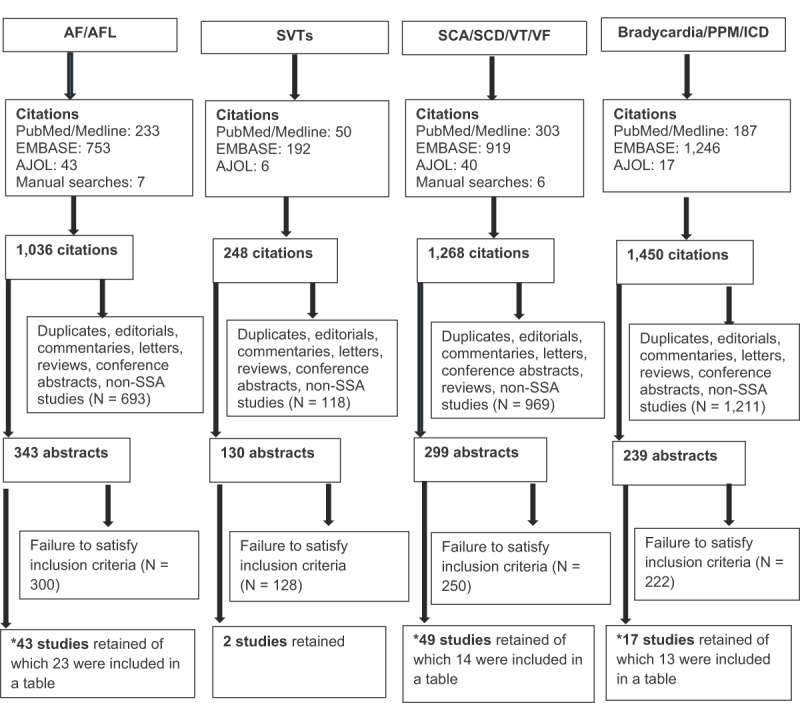
Systematic search for atrial arrhythmias, sudden cardiac arrest/sudden cardiac death & ventricular arrhythmias, bradycardia, and cardiac implantable electronic devices in sub-Saharan Africa. AF/AFL (atrial fibrillation/atrial flutter); AJOL (African Journals Online); ICD (implantable cardioverter defibrillator); PPM (permanent pacemaker); SVTs (supraventricular tachycardias); SCA (sudden cardiac arrest); SCD (sudden cardiac death); SSA (sub-Saharan Africa); VT (ventricular tachycardia); VF (ventricular fibrillation). Initial search restricted to human studies published up to March 31^st^, 2019. *Studies not included in tables had relevant findings, but were too heterogenous to fit into a table.

We systematically searched the PubMed/MEDLINE, Excerpta Medica Database (EMBASE), and African Journals Online (AJOL), to identify all relevant studies published until March 31^st^, 2019 and restricted to humans, reporting on cardiac arrhythmias in SSA, without language restriction. The search strategy and terms used were as follows: 1) Atrial fibrillation OR atrial flutter AND Africa; 2) supraventricular tachycardia OR atrioventricular nodal reentry tachycardia OR atrial tachycardia OR atrioventricular reentry tachycardia OR Wolff-Parkinson-White syndrome AND Africa; 3) sudden cardiac arrest OR sudden cardiac death OR ventricular arrhythmia OR ventricular tachycardia OR ventricular fibrillation AND Africa; and 4) bradycardia OR pacemaker OR defibrillator OR cardiac implantable electronic devices AND Africa. Sub-Saharan African studies were then filtered from the identified studies. Inclusion criteria for AF/AFL and SVTs were studies reporting prevalence, risk factors, arrhythmia treatment, oral anticoagulation, and follow-up outcome. Inclusion criteria for SCA/SCD were studies reporting out-of-hospital cardiac arrest (OHCA) or in-hospital cardiac arrest (IHCA), attempted cardiopulmonary resuscitation (CPR), return of spontaneous circulation (ROSC), and survival. Manual searches of references of published articles were also undertaken. We excluded editorials, commentaries, letter, notes, conference abstracts without full published articles, and narrative reviews (See Figure 1). Data extraction and quality assessment were meticulously done according to set criteria by two authors (MFY & MN) independently. The marked heterogeneity of studies among specific arrhythmia entities precluded any meta-analysis.

## Results

As shown in Figure 1, the initial PubMed/Medline, EMBASE, and AJOL search restricted to humans till March 31^st^, 2019 and manual searches of references of published articles for AF/AFL, revealed 1,036 citations. When narrowed to SSA with exclusion of duplicates, editorials, letters, reviews, conference abstracts without full article publications, and commentaries, they were 343 remaining abstracts to screen, of which 43 made the inclusion criteria and 23 studies had compatible data to be entered in Table 1. For SVTs, the final number of abstracts reviewed was 20, and only two studies were suitable. The search for SCA, SCD, and ventricular arrhythmias, revealed 1,268 citations. After applying the exclusion criteria, there were 299 abstracts left to screen, of which 49 made the inclusion criteria, and 14 studies had compatible data to be entered in Table 2. The search for articles on bradycardia and cardiac implantable electronic devices (CIEDs) revealed 1,450 citations, which were narrowed down to 239 abstracts via exclusion criteria (Figure 1). After a detailed review, 17 studies met inclusion criteria and 13 of these studies had compatible data which was entered in Table 4.

**Table 1:** Atrial fibrillation studies in sub-Saharan Africa.

Author, Year & Country	Mean age in years	Study size & population	Gender	Prevalence of AF	Comorbidities	AAM or rate-control medication	CHADS2 ≥ 1 or CHA2DS2 VASC ≥ 2, mean or median	OAC*	FU & Mortality

**Mwita [49]** **2019** **Botswana**	66.7	138; Tertiary hospital	Females 63.8% Males 36.2%	–	HTN 59% RHD 38% HF 36% CVA/TIA 22% DM 8% Obesity 35%	Digoxin 32% BB 70% Amiodarone 0% CCB 0%	Mean CHA2DS2 VASC 3.6	73.8% (Warfarin 69%) in CHA2DS2 VASC ≥ 2	12 months mortality 14.5%
**THESUS-HF** **Registry [22]** **2018** **Multinational** **(9 SSA countries)**	57	206/1006; Heart failure	Females 53.1% Males 46.9%	21.8%	HTN 52% HF 100% VHD 44% CM 38% CAD 5.3% CVA 3.4%	–	–	Admission 52.2% 6 months FU 21.8%	16% rehospitalization or death at 60 days follow-up
**Thomas [50]** **2018** **South Africa**	–	42; Tertiary Hospital	Females 43.0% Males 57.0%	Paroxysmal 50% Persistent 29% Permanent 12% Atrial flutter 17%	–	–	–	–	5.8 years CVA 10% CM 17%
**Muthalaly [51]** **2018** **Uganda**	42	0/856; Rural community	Females 62.5% Males 37.5%	0%	HTN 11.6% DM 3.4% CAD/HF 5.6% CVA 2.7%	–	–	–	–
**Temu [48]** **2017** **Kenya**	37 NVAF 69 VAF	77 VAF/69 NAVF; Clinic and hospitalized patients	Females 67.1% Males 32.9%	-	VAF/NVAF HTN 29%/73% HF 10%/49% RHD 100%/0% CVA 23%/26% DM 1%/8.7%	BB 49% Digoxin 36%	Mean CHADS2 score 2.2 for NVAF	79% for NVAF TTR 52% VAF, 56% NVAF	12 months mortality 10% VAF, 15% NVAF
**Greffie [52]** **2016** **Ethiopia**	67.4	94; Stroke patients	Females 53.1% Males 46.9%	28.7% (Ischemic 34% Hemorrhagic 14%)	–	–	–	–	Hospital case fatality with AF 22.2% & without AF 8%. 12 months mortality 14.5%
**Lugero [43]** **2016** **Uganda**	52	102, Cardiology Unit	Females 56.9% Males 43.1%	–	HTN 50% HF 50% RHD 32% Obesity 10% ICVA 12.8%	–	74.5%	–	In hospital mortality 9.8%
**Yameogo [45]** **2016** **Burkina Faso**	65	103/970; Cardiology department	Females 44.6% Males 55.4%	10.6% NVAF 66% Paroxysmal 11.8% Persistent 70.6% Permanent 17.6%	HTN 66.2% HF 86.8% ICVA/TIA 33.8% VD 20.6% DM 20.6% ↑TSH 10.3%	–	97% Median CHA2DS2VASC score = 3.9	35.3%	–
**Ajayi [53]** **2016** **Nigeria**	67	55/1462; Tertiary referrals	Females 47.3% Males 52.7%	3.8%	HTN 87.3% HHD 65.5% DCM 16.4% CVA 40% COPD 25% DM 18% ↑TSH 4.4%	–	65.5%	–	–
**Akpa [44]** **2015** **Nigeria**	60	68/228 cardiology unit/clinic	Females 42.6% Males 57.4%	28.9% VAF 14.7% NVAF 85.3%	HHD 58.8% DCM 19.2% RHD 14.7%	Digoxin 92.6% Amiodarone 6%	100%	8.8%	-
**Mandi [40]** **2015** **Burkina Faso**	63	69 NVAF/159 ICVA patients	Females 62.3% Males 37.7%	43.3% Paroxysmal 13% Persistent 52% Permanent 35%	HTN 85% DM 21.7% Prior ICVA 17.4%	Digoxin 7.3% BB 27.5% Amiodarone 20.3%	100% Mean CHA2DS2VASC score = 4.7	52%	21.7% in-hospital mortality
**RE-LY Registry [41] (baseline data)** **2014** **Multinational** **(included 10 SSA countries)**	57	1137 (SSA only); Emergency presentations	Females 53.1% Males 46.9%	Paroxysmal 8.9% Persistent 9.6% Permanent 81.4%	HF 63.8% HTN 54% All VHD 32.6% RHD 21.5% CAD 5.5% DM 14% CVA/TIA 14.1%	BB 21.7% Digoxin 34.5% CCB 2.0% Amiodarone 3.3%	Mean CHADS2 score 1.8	19.4% TTR 32.7%	–
**Jardine [39]** **2014** **South Africa**	67	302; National Registry	Females 40.1% Males 59.9%	Paroxysmal 32.1% Persistent 21.2% Permanent 46.7%	HTN 65.9% HF 32.5% VHD 27.5% CAD 26.8% DM 15% ICVA/TIA 13.6%	Rate-control 63.9% Rhythm-control 36.1% BB 59.6% CCB 13% Class IC 3% Class III 33.8% DCCV 13.2% Catheter ablation 4.2%	Mean CHA2DS2VASC score = 3.08	75.2%	–
**REMEDEY [24]** **2016** **Multinational (included 11 SSA countries)**	28	586/3343 (all study population); RHD	Females 66.1% Males 33.9%	18–28% depending on income-level	RHD 100%	–	40.7%	69.5% TTR 27.4%	2 years mortality 16.9%
**Koopman [15]** **2014** **Ghana**	66	924; Rural population	Females 48.1% Males 51.9%	0.3%	HTN 24% CAD 1.2%	–	–	–	–
**Shavada [37]** **2013** **Kenya**	67	162; Discharge diagnosis	Females 44.0% Males 56.0%	Paroxysmal 40% Persistent 20% Permanent 40%	HTN 68% HF 38% DM 33% CAD19% VHD 12%	Rate-control 78% Rhythm-control 22% BB 46% Digoxin 44% CCB 9% Amiodarone 10% DCCV 8%	78%	72%	6months mortality 6.5%
**Coulibaly [20] 2013** **Mali**	55	111/3964; cardiac admissions	Females 48.0% Males 52.0%	2.8% Permanent 73%	HHD 33% VAF 33% CAD 12.5% CVA 20%	Digoxin 21% BB 51% CCB 1% Amiodarone 13% DCCV 0%	78.6%	22.9%	
**Dewhurst [16]** **2012** **Tanzania**	78	15/2232; Community	Females 56.3% Males 43.7%	0.67%	–	–	–	–	One-year mortality 53%
**Ntep-Gweth [38]** **2010** **Cameroon**	66	172; Office visit	Females 56.4% Males 43.6%	Paroxysmal 23% Persistent 22% Permanent 56%	HTN 65% HF 58% HHD 48% RHD 26% CM 16% DM 10% CAD 6% ICVA 16.1%	Rate-control 84% Rhythm control 16% BB 11% Digoxin 62% CCB 9% Amiodarone 29% DCCV 2.3%	91.9%	34.2%	29.5% died during 11 months of follow-up; 16.1% CVA
**Sliwa [18]** **2010** **South Africa**	59	246/5328; Cardiac admissions	Females 44.0% Males 56.0%	4.6%	HF 56% HTN 60% HHD 47% VHD 44% RHD 21% CM 15% CAD 6.5% Alcohol 48%	BB 36% Digoxin 24% Amiodarone 7.3%	–	33%	–
**Mbaye [19]** **2010** **Senegal**	57	150; Cardiac admissions	Females 68.7% Males 31.3%	5,4%	HHD 41% VHD 37% CM 4.7% ICVA 14.7% CAD 2.7%	Rate-control 87% Amiodarone 7% DCCV 1.3%	–	62%	–
**Coulibaly [17] 2010** **Ivory Coast**	59	217/3908; Cardiac admissions	Females 64.8% Males 35.2%	5.5%	HF 63% HHD 48% RHD 28%	–	47%	–	–
**Bhagat [54]** **1999** **Zimbabwe**		200; Cardiology clinic	–	–	–	–	79% urban 83% rural	38% urban 19% rural	–

AAM (antiarrhythmic medication); BB (betablocker); CAD (coronary artery disease); CCB (non-dihydropyridine calcium channel blocker); CM (cardiomyopathy); COPD (chronic obstructive pulmonary disease); CVA (cerebrovascular accident); DCCV (direct current cardioversion); DM (Diabetes mellitus); FU (follow-up); HF (heart failure); HHD (hypertensive heart disease); HTN (hypertension); ICVA (ischemic cerebrovascular accident); NVAF (non-valvular atrial fibrillation); OAC (oral anticoagulation); RHD (rheumatic heart disease); TIA (transient ischemic attack); ↑TSH (hyperthyroidism); TTR (time in therapeutic range); VAF (valvular atrial fibrillation); VD (vascular disease); VHD (valvular heart disease). * Percentage of patients with CHADS2 ≥ 1 or CHA2DS2VASC ≥ 2 who were anticoagulated.

**Figure 2:**
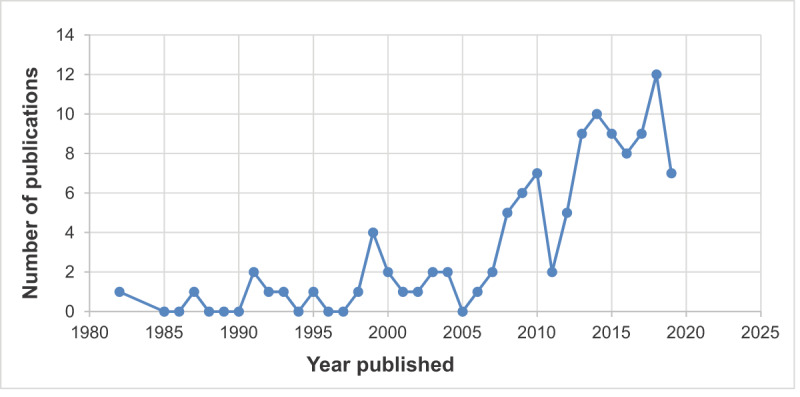
Trend of publications on cardiac arrhythmias from 1980 to March 31^st^, 2019 in sub-Saharan Africa.

Figure 2 shows an encouraging increasing trend of publications on cardiac arrhythmias in SSA over the past four decades, based on the studies identified for this systematic review.

## Atrial fibrillation/atrial flutter (AF/AFL)

### Prevalence and risk factors

Table 1 summarizes key findings from hospital-based and community-based AF/AFL studies in SSA. AF prevalence is low in the general population of SSA at <1% and increases with age [3,15,16], 3–7% in hospital cardiology admissions or newly diagnosed cardiovascular diseases [17,18,19,20,21], 16–22% in heart failure patients [22,23], 10–14% in newly diagnosed and 18–28% in established rheumatic heart disease (RHD) patients [24,25,26], 25% in patients with tuberculous pericarditis [27], 6% *de novo* cases post-cardiac surgery [28], 9.5% in pregnant women with structural heart disease [29], 2–10% of *de novo* stroke patients [30,31,32,33], and varies between 25–65% in patients attending oral anti-coagulation clinics in SSA [34,35,36]. In SSA there is a high proportion of permanent AF (12–81.4% across studies) and persistent AF (9.6–70.6%), compared to prevalence of paroxysmal AF (8.9–50%) [20,37,38,39,40,41] as shown in Table 1. Prominent risk factors or comorbidities associated with AF/AFL in SSA are hypertension, which is observed in 50–87% of cases, heart failure 32–64%, diabetes 4–63%, RHD 15–38%, dilated cardiomyopathy 16–38%, stroke 3–40%, and CAD 1.2–26% of AF/AFL patients (Table 1). Other AF risk factors include non-rheumatic valvular diseases, smoking, obesity, obstructive sleep apnea, hyperthyroidism, COPD, congenital heart disease, and increased alcohol intake [15,18,19,20,28,37,38,41,42,43,44,45]. There appears to be a female preponderance of AF/AFL in SSA with studies showing 40–69% of patients being females versus 31–60% males (Table 1). Studies in this region have shown that the presence of AF/AFL is associated prospectively with significantly high mortality (15–53%), increased rates of heart failure hospitalization, and non-fatal cardioembolic strokes during follow-up (10–15%) [19,38,46,47].

### Anticoagulation

Use of oral anticoagulation (OAC) in AF/AFL patients in SSA was noted to be very variable from 9–79% in patients with CHA2DS2VASC score ≥2 or CHADS2 score of ≥1 across studies, as shown in Table 1. In patients from SAA who were anticoagulated with Vitamin K antagonists, average time in therapeutic range (TTR) calculated by the Rosendaal method was noted to be generally low at 27–56% [24,41,48]. Vitamin K antagonist (VKA) oral anticoagulant were available in all countries surveyed recently by Pan African Society of Cardiology (PASCAR), while non-VKA oral anticoagulants (NOACs) were less available as follows: rivaroxaban (available in 90% of countries), dagibatran (45%), apixaban (22%), and endoxaban (0%) [14].

## Supraventricular tachycardias (SVTs)

From the initial citations for SVTs, only two studies all from South Africa met inclusion criteria as shown in Figure 1 [55,56]. Among a pediatric population, the differential diagnoses of SVT were atrioventricular nodal reentrant tachycardia (AVNRT) 51%, atrioventricular reentrant tachycardias (AVRT) 24%, atrial tachycardia (AT) 22%, and junctional ectopic tachycardia 3% [55]. In the other study, nine patients with Wolff-Parkinson-White syndrome and symptomatic paroxysmal SVTs had their accessory pathways successfully surgically divided without complications or recurrence (four posteroseptal, three left free wall, and two right free wall accessory pathways) [56].

## Sudden cardiac arrest/sudden cardiac death

### Epidemiology and rhythm of arrest

The key finding of this systematic review is the sparsity of studies on SCA/SCD in this region. Table 2 depicts the few studies on SCA/SCD in SSA [57,58,59,60,61,62,63,64,65,66,67,68,69,70]. From the published studies, one of the salient findings is the low mean age of SCA/SCD with a range of 35–60 years across studies among adults, with higher rates in males compared to females in majority of studies. The reported incidences of OHCA range from 6–34 per 100,000 inhabitants in SSA [59] [65]. The incidence of IHCA among cardiology admissions is approximately 6% in this region [61]. The most common underlying rhythm of SCA/SCD in SSA is asystole, followed by pulseless electrical activity (PEA), then VT/VF, and unknown [58,65].

**Table 2:** Sudden cardiac death/sudden cardiac arrest studies in sub-Saharan Africa.

Author, Year, country	Mean age in years or age range	Sample size	Gender	Study population	CPR attempted	Rhythm of arrest	ROSC	Etiologies & Comorbidities	Survival to discharge

**Edwards-Jackson et al [57], 2019, Malawi**	30 days to 13 years	135	–	Paediatric population IHCA	100%	–	6%	Malaria 51%	0% (100% mortality)
**Ngunga et al [58], 2018, Kenya**	61	353	Females 46.5% Males 53.5%	IHCA	Not mentioned	Asystole 47.6%, PEA 38.2%, VT/VF 5.4%, Unknown 8.8%	Asystole patients 17.3%, PEA 40.7%, VT/VF 57.9%, Unknown 25.8%. Mean time to ROSC 5.3 mins	Heart Failure 9.1% HTN 39.7% DM 25.5% CAD 6.0% CVA 4.9% Cancer 9.1% HIV/AIDS 14.5% Sepsis 19%	4.2%
**Bonny et al [59], 2017, Cameroon**	Men 36 Women 35	27/288 Incidence of SCD 33.6 per 100 000 person-years	Females 48.1% Males 51.9%	OHCA 63%	3.7%	–	–	Heart failure 14.8% HTN 22.2% DM 11.1% CAD 7.4% HIV 7.4% Tropical disease 3.7%	–
**Adekola et al [60], 2016**, **Nigeria**	1–18 years 23.33% >18years 77.67%	60/4,229 cases	Females 55.0% Males 45.0%	Perioperative cardiac arrests	100%	-	**56.7%**	No co-existing disease 81.2% HTN 13.3% DM 8.3% Sickle cell 1.6% Incidence in ASA III/IV/V >ASA I/II	20.6%
**Talle et al [61], 2015**, **Nigeria**	49 all patients, 39 years SCD patients	Total 388 cardiac admissions, 56 deaths, 23 (41.1%) SCD	Females 52.2% Males 47.8%	Cardiac admissions	52.1%	–	8.3%	Heart failure 82.6% CAD 39.1% Peripartum CM 21.7% DCM 17.4% RHD 17.4% HTN 47% DM 21% PHTN 4.3%	–
**Akinwusi et al [62], 2013, Nigeria**	46	29/718 (4%)	Females 13.8% Males 86.2%	Adult medical deaths	–	–	–	CVD 51.7% (HHD 86.7%, HF 80%) Respiratory 20.7% PE 10.4% CNS disease 13.8% GI 13.0% Chemical/drug 13.8% HTN 48.3%	–
**Tiemensma et al [64], 2012**, **South Africa**	42.6	816	Females 31.0% Males 69.0%	Sudden and unexpected adult deaths	–	–	–	CVD 17.2% (CAD 75.7%) Respiratory 15.0% CNS 7.5% GI 2.9% GU 1.2%	
**Kwari et al [63], 2010**, **Nigeria**	–	14/4,015	–	Perioperative cardiac arrest	–	–	–	Patients with ASA class III/IV risk status suffered more arrest than ASA I/II	14%
**Stein et al [65], 2009**, **South Africa**	Adults	510	–	OHCA	40% (By-stander 36%) Median response time 9 minutes	VT/VF 23%. Only predictor of ROSC was shockable rhythm	18%	Cardiac causes 75%	–
**Olotu et al [66], 2009**, **Kenya**	28 months	114	Females 40.0% Males 60.0%	IHCA Pediatric patients	100%	–	26%	Malaria Septicemia Severe malnutrition	16%
**Rotimi et al [67], 2004**, **Nigeria**	Men 53.7 Women 52.2	79	Females 25.3% Males 74.7%	Medico-legal autopsies	–	–	–	HHD 83.5% CAD 6.3%	–
**Schneider et al [69], 2001, Ethiopia**	–	92	–	Sudden unexpected deaths based on police reports	–	–	–	CAD 47.8% RHD 7.6%	–
**Rotimi et al [68], 1998**, **Nigeria**	28–80 years	50	Females 30.0% Males 70.0%	Coroner’s autopsies	–	–	–	HTN 82% Heart failure 68% CAD 4%	–
**Arthur et al [70], 1995**, **Ghana**	–	16	–	Pediatric patients	–	–	–	Cardiac disease 50% Sickle cell 6.25% Tuberculosis 6.25% No chronic disease 37.5%	–

ASA (American Society of Anesthesiologists); CAD (coronary artery disease); CNS (central nervous system); CPR (cardiopulmonary resuscitation); CVD (cardiovascular disease); DCM (dilated cardiomyopathy); DM (diabetes mellitus); GI (gastrointestinal); GU (genitourinary); HHD (hypertensive heart disease); HIV (human immunodeficiency virus); HTN (hypertension); IHCA (in-hospital cardiac arrest); OHCA (out-of-hospital cardiac arrest); PE (pulmonary embolism); PEA (pulseless electrical activity); PHTN (pulmonary hypertension); RHD (rheumatic heart disease); ROSC (return of spontaneous circulation); SCD (sudden cardiac death); VT/VF (Ventricular tachycardia/ventricular fibrillation).

### Reported underlying etiologies of SCA/SCD and ventricular arrhythmias

The reported underlying etiologies of SCA/SCD and ventricular arrhythmias in SSA are shown in Table 3. Studies have identified *hypertensive heart disease, coronary disease, cardiomyopathy, and valvular heart* disease, especially *rheumatic heart disease*, in heterogeneous orders, as the most common causes of SCA/SCD among adults in SSA [61,64,67,69,71,72,73], while malaria was the prominent cause among the pediatric victims [57,66]. Malignant arrhythmogenic inherited diseases have been identified in Blacks in SSA. Cases of *hypertrophic cardiomyopathy* have been reported in Black Africans [74,75]. Little was known about *arrhythmogenic right ventricular cardiomyopathy (ARVC)* in SSA, but registry data from South Africa revealed similar clinical presentation and an annual SCA/SCD rate comparable to other large registries from the Western World [76,77]. *Brugada syndrome* is associated with SCD [78,79]. *Loss-of-function CACNA1C variant*, Cavα1c-T1787M, present in 0.8% of the Black African population, has recently been identified as a new risk factor for *ventricular arrhythmias* [80]. Although electrocardiographic patterns of *early repolarization* (ER) have been reported in a Black African population [81], their correlation with malignant arrythmias or SCA/SCD have not been studied in SSA. Notwithstanding, studies in African Americans have shown that the relatively high prevalence of ER in this population is not independently predictive of adverse outcomes [82,83]. *Ventricular non-compaction*, a prominent cause of VT/VF, has been identified in patients of African ancestry with the prevalence of 6.9% in one cardiomyopathy clinic in South Africa [84], and in other SSA studies [85,86]. Apart from one case report in a black infant [87], no other studies have documented congenital long QT syndrome in Black Africans. For example, a congenital long QT series of 41 patients in South Africa had Blacks (0%), Whites (87.8%), mixed race (9.8%), and Indian (2.5%) [74], and one other study still in South Africa did not mention ethnicity [88]. *Acquired long-QT* syndrome in heart failure cohorts has been associated with excess mortality [89,90]. In patients with peripartum cardiomyopathy, prolonged corrected non-congenital QT interval and sinus tachycardia on baseline ECG were independent predictors of poor composite outcome which included death during follow-up [90].

**Table 3:** Reported underlying etiologies of sudden cardiac arrest/sudden cardiac death among adults in sub-Saharan Africa.


Cardiomyopathies [58,61]
Hypertensive heart disease [58,62,67]
Coronary artery disease [58,59,61,64,69]
Rheumatic heart disease [61,69]
Congenital heart disease [96]
Arrhythmogenic right ventricular cardiomyopathy [76,77]
Hypertrophic cardiomyopathy [74,75]
Brugada syndrome [78,79]
Congenital Long QT syndrome (seen only in non-Black populations) [74,88,101]
Ventricular non-compaction [84]
Pulmonary embolism [62,91]
Endomyocardial fibrosis [95]
Pulmonary hypertension [61]
Pericarditis (mainly tuberculous) [92]
Aortic dissection/rupture [64]
Endemic parasitic infections like trypanosomiasis & schistosomiasis [9,59,93]
Sarcoidosis [102,103,104]
Respiratory disease [58,62,64]
Septicemia [58,66]
HIV/AIDS [58,59]
Cancer [58]
Tuberculosis [58,64,70]
Renal disease [58,64]
Liver disease [58,64]


*N/B:* Detailed investigations for the cause of SCA/SCD are sparse in SSA. Therefore, uncertainty remains about the relative frequencies of these underlying etiologies.

Reports of SCA/SCD due to *pulmonary embolism* [62,91], *pulmonary hypertension* [61], and *aortic dissection/rupture* [64] are seen. *Pericarditis*, especially tuberculous which accounts for about 65–91% of all pericarditis cases in SSA, is associated with premature death [92]. Some *endemic parasitic infections* have been identified as potential causes of arrhythmias and conduction abnormalities. These include trypanosomiasis-induced cardiomyopathy through chronic pan-carditis (*Trypanosoma brucei* which is of the same genus as *Trypanosoma cruzi* which causes Chagas disease in Latin America), and schistosomiasis-induced pulmonary hypertension leading to right sided cardiomyopathy plus arrhythmias, amebiasis, toxoplasmosis, among others [9,93]. There have been case reports of SCA/SCD events with use of an antimalarial, halofantrine [94]. *Endomyocardial fibrosis* which is endemic in SSA has very poor prognosis with survival after diagnosis reported to be two years due to malignant arrhythmias, heart failure, and thromboembolism [95]. SCA/SCD due to *congenital heart disease* has been reported in SSA [96]. Short QT syndrome, catecholaminergic polymorphic ventricular tachycardia (CPVT), and other VTs forms have not been observed in the SSA literature.

### Cardiopulmonary resuscitation (CPR)

As shown in Table 2, there is an alarming observed gross lack of CPR awareness among the SSA populations. CPR was only attempted in 3.7–40% of OHCA and only attempted in about 50% of IHCA cases among adults [59,61,65], in non-perioperative studies. Excluding perioperative cardiac arrest, ROSC was achieved in <20% of adult SCA cases and survival to discharge was low at <5%. The best predictor of ROSC was a shockable rhythm (VT/VF), followed by PEA, with asystole having the worse outcomes [58,65]. Multiple surveys in SSA have demonstrated that even clinicians including physicians do not have adequate basic life support (BLS) and advanced cardiac life support (ACLS) training (about half of those surveyed), and the majority are unable to operate an automated external defibrillator (AED) [13,97,98]. In South Africa where EMS services are available, overall knowledge and skill performance of CPR is still well below standard by EMS personal with only 25% of the required standards met [99]. In addition to lack of optimal resuscitative measures, quality improvement schemes are also deficient in SSA. A survey of 17 hospitals in SSA found that only 20% of these had a cardiac arrest response team system, only 21% documented CPR events, and only 21% reviewed such events for education and quality improvement [100].

## Bradyarrhythmias and cardiac implantable electronic devices (CIEDs)

### Indications of device implantation, type of device

Seventeen studies on bradyarrhythmias and CIEDs in SSA were identified through the systematic search [9,11,12,105,106,107,108,109,110,111,112,113,114,115,116,117,118,119] (Figure 1). Table 4 depicts 13 of these studies with some uniform data that could be organized into one table. The commonest indication for permanent pacing in SSA is atrioventricular block (AVB) accounting for 45–100% of all cases across studies, compared to sick sinus syndrome at 0–35%, and others (atrioventricular node ablation, cardiac re-synchronization therapy, etc) 0–20%. Single chamber ventricular (VVI) pacemakers are the most frequently implanted (17–87%), compared to dual chamber (12–82%), and others like atrial-sensed ventricular-paced (VDD) (0–15%) [107,108,109,110,111,112,113,114,115,116,117,120]. Cost constraints have been identified as the reason for high implant percentage of VVI compared to DDD [114].

**Table 4:** Cardiac implantable electronic devices in sub-Saharan Africa.

Author, Year, country	Mean age in years	Sample size	Gender	Indication	Types of CIEDs	Chamber of implantation	Complications

**Tchoumi et al [108], 2019, Cameroon**	62	130	Females 40.0% Males 60.0%	SSS 29.1% AVB 88 70.9%	PPM 124 ICD 4 CRT 2	VVI 17.0% DDD 81.5% CRT 1.5%	- Pocket infection 4 (3.1%) - Lead displacement 4 (3.1%) - Pneumothorax 2 (1.5%) - Hemothorax 2 (1.5%)
**Adoubi et al [109], 2018, Ivory Coast**	67	283	Females 50.9% Males 49.1%	SSS 17% AVB 83%	PPM	–	–
**Jouven et al [110], 2016**, **14 SSA countries**	–	502 during 16 missions to SSA	–	–	PPM	–	- No periprocedural complications - 52% of patients initially listed as suitable died before the missions arrived
**Jama et al [107], 2015, South Africa**	-	126	Females 52.9% Males 47.1%	*PPM* SSA 12.8% AVB 79.4% Others 7.8% *ICD* Secondary prevention 79.2% Others 20.8%	*PPM 102* New 50% Recycled 50% *ICD 24* New 50% Recycled 50%	*PPM* VVI 79.5% DDD 17.6% Others 2.9% *ICD* VVI 100%	- No device infection, malfunction, early battery depletion or device removal in either the re-used or new devices groups
**Ikama et al [111], 2015, Congo**	70	8/20 implanted	Females 50.0% Males 50.0%	AVB 100%	PPM	–	- No complications - 8 patients (40%) of the initial 20 died before mission arrived
**Falase B et al [112], 2013, Nigeria**	68	51	Females 43.1% Males 56.9%	SSS 9.8% AVB 90.2%	PPM	VVI 56.9% DDD 43.1%	- Infection 3 (5.9%) - Lead displacement 3 (5.9%) - Pocket erosion 2 (3.9%) - Death 1 (2%)
**Kane et al [113], 2012, Senegal**	66	107	–	–	PPM	–	- Infection 5.6%
**Ekpe et al [119], 2008, Nigeria**	70	23	Females 48.0% Males 52.0%	SSS 0% AVB 100%	PPM	Endocardial 65% Epicardial 35%	–
**Thiam et al [116], 2003, Ivory Coast**	–	92	Females 48.9% Males 51.1%	–	*PPM* New 47% Recycled 53%	VVI 87% DDD 23%	- Infection 5 (5.4%) - Lead displacement 3 (3.3%) - Pacemaker syndrome 1 (1.1%) - Death 1 (1.1%)
**Millar et al [114], 2001, South Africa**	–	1643	–	*Public hospitals* SSS 16.2% AVB 75.3% AVNA 3% Others 6.3% *Private hospitals* SSS 34.9% AVB 45.3% AVNA 13.6% Others 7.6%	PPM	*Public hospitals* AAI 0.4% VVI 73% VDD 14.5% DDD 12.1% *Private hospitals* AAI 0.4% VVI 53.3% VDD 10.9% DDD 42.3%	–
**Diop et al [117], 2000, Senegal**	54	12	Females 41.7% Males 58.3%	–	PPM	VVI 41.7% DDD 58.3%	- Pocket infection 2 (16.0%)
**Mayosi et al [115], 1999, South Africa**	21–50	232	Females 41.8% Males 58.2%	SSS 25% AVB 62% Others 13%	PPM	VVI 65% DDD 35%	–
**Dos Santos et al [118], 1982, South Africa**	17–78	57	Females 61.0% Males 39.0%	SSS 4% AVB 91% SSS + AVB 5%	PPM	VVI 98.3% DDD 1.7%	- Infection 2 (3.5%) - Lead displacement 2 (3.5%) - Erosion 3 (5.3%)

AAI (single chamber atrial pacemaker); AVB (atrioventricular block); AVNA (atrioventricular node ablation); CIEDs (cardiac implantable electronic devices); CRT (cardiac resynchronization therapy); DDD (dual chamber pacemaker); ICD (implantable cardioverter defibrillator); LVEF (left ventricular ejection fraction); PPM (permanent pacemaker); SSA (sub-Saharan Africa); SSS (sick sinus syndrome); VDD (dual chamber sensing, ventricular pacing pacemaker); VVI (single chamber ventricular pacemaker).

### Implantation rates of pacemakers and defibrillators plus complications

Epidemiological survey data emanating from SSA indicate that there are still countries without a CIEDs implanting center, and a patchy presence in others. The first report of the PASCAR on the statistics of the use of CIEDs and ablation procedures revealed that 26% of the 31 countries surveyed did not perform any permanent pacemaker (PPM) implantations. The median pacemaker implantation rate was 2.66 per million population per country, median number of PPM implantation centers was 0.14 per million inhabitants and 0.10 operators per million population. Implantable cardioverter-defibrillator (ICD) and cardiac resynchronization therapy (CRT) were performed in 39% and 48% countries respectively, mostly by humanitarian visiting cardiac teams from abroad. Rates of centers performing ICD and CRT were similar and ranged from 0.02 to 1.59 per million population [11,12]. In a more recent second report of PASCAR survey (2011–2018), 18% of countries in this region still did not perform PPM implantations, and implantation and operator rates rate were 2.79 and 0.772 per million population respectively in implanting countries. ICD and CRT were performed in 65% and 52% countries respectively, while reconditioned CIEDs were used in 22% countries [14]. In a study with long-term survival data after permanent pacing in SSA, there was a 17% mortality after a median follow-up time of about nine years [115]. Complications of pacing across studies are infections (0–6%), lead displacements (0–6%), pneumothorax (0–1.5%), hemothorax (0–1.5%), erosions (0–5.3%%), and death (0–2%) [107,108,109,110,111,112,113,114,115,120].

## Diagnostic tools for arrhythmias

Electrocardiography (ECG) is available in all SSA countries, 2-D echocardiography in 87%, Holter ambulatory cardiac monitoring in 74%, exercise tolerance test in 52%, tilt table test in 13%, cardiac computed tomography and cardiac magnetic resonance imaging available in <25% of countries, and signal average ECG is done only in South Africa [9,11]. Electrical cardioversions are only done in 45% of SSA countries [12].

## Antiarrhythmic medications

Recent survey of countries in Africa by PASCAR showed that digoxin and amiodarone were available in all surveyed countries, flecainide (80% of countries), sotalol (75%), propafenone (22%), quinidine (17%), and mexiletine (4% of countries) [14], and prior surveys showed that atropine and intravenous lidocaine were also present in some African countries [9,11]. These findings were also observed in individual AF/AFL studies where betablockers and non-dihydropyridine calcium channel blockers are prescribed and dispensed in this region [18,20,37,38,39,41,44,48]. There is no available information about use of adenosine to manage acute SVTs in this region.

## Electrophysiological studies and ablations

**Figure 3:**
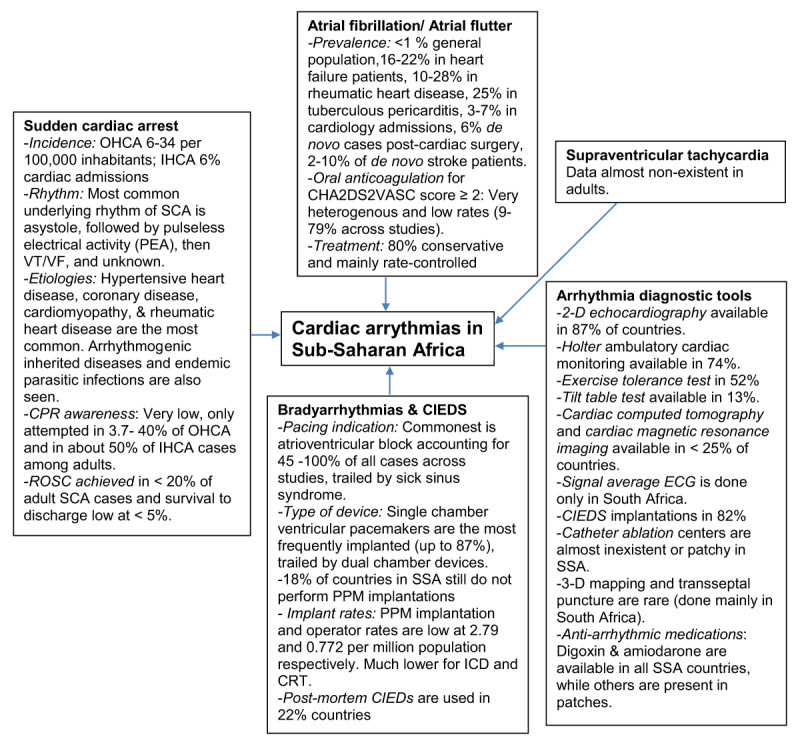
Central illustration of cardiac arrhythmias in sub-Saharan Africa. CIEDs (cardiac implantable electronic devices); CPR (cardiopulmonary resuscitation); CRT (cardiac resynchronization therapy); ICD (implantable cardioverter defibrillator); IHCA (in-hospital cardiac arrest); PPM (permanent pacemaker); OHCA (out-of-hospital cardiac arrest); ROSC (return of spontaneous circulation); SSA (sub-Saharan Africa); VT/VF (ventricular tachycardia/ventricular fibrillation).

Management of arrhythmias in SSA is largely non-invasive as electrophysiological (EP) study and catheter ablation centers are almost inexistent or patchy in SSA. South Africa is the only country in this region where complex ablations requiring 3-D mapping and transseptal puncture are performed [11,12,14]. Even in South Africa, national AF registry data showed that only 4.2% of AF patients underwent catheter ablations [39]. About 80% of AF/AFL patients are managed with rate control strategy across studies in SSA [18,20,37,38,41,44]. Figure 3 summarizes cardiac arrhythmias in SSA.

## Discussion

### Atrial fibrillation/atrial flutter

We observed that the prevalence of AF/AFL is <1% in the general population in sub-Saharan Africa. There are estimated 1.3 million people with AF/AFL in SSA and according to the 2017 Global burden of Disease Study, this region has one of the lowest prevalence rates of AF/AFL at 0.13%, compared to ~1.5% in the high-income countries (HIC) of Western Europe and North America, but does increase with age [3]. Given lack of resources including ECG and ambulatory cardiac monitoring as well as the high prevalence of ‘highly arrhythmogenic’ conditions/risk factors/circumstances (e.g. RHD, cardiomyopathies, pericardial disease, unavailability of surgery, etc…), this low prevalence in SSA could represent an under-estimate. Other important factors include lack of healthcare access and survivorship bias i.e. lower life expectancy with communicable diseases. AF/AFL occurs at younger ages in SSA as seen in the RE-LY global registry, where many patients from SSA were younger (average age 57 years) compared to Western World (70 years) [41]. Risk factors of AF are similar in SSA compared to HIC, except the significantly higher prevalence of rheumatic heart disease in SSA (22%) vs HIC (~2%) in AF patients observed in one large registry, as well as lower rates of ischemic heart disease in SSA (6%) vs HIC (~18%) [41], and high AF prevalence 25% in pericarditis in SSA vs 4.3% seen in pericarditis in HIC [121]. The findings of this systematic review are similar to those of prior reviews in Africa with respect to AF prevalences rates, risk factors, and co-morbidities [8,122].

Despite the relatively lower prevalence of AF in people of African descent, the presence of AF is associated with higher rates of strokes, heart failure, and mortality compared to Caucasians, and Black patients with AF are much younger than patients of other races [123]. Atrial fibrillation was seen in 43–82% of patients with cardio-embolic strokes in SSA [40,46]. Uncontrolled hypertension, low use of anticoagulation due less access and high costs (especially non-vitamin K-dependent oral anticoagulants) and poor time in the therapeutic range among patients on vitamin K-dependent oral anticoagulants, low use of heart failure medication, and late presentation with complications are plausible reasons for this increased risk of adverse outcomes. While anticoagulation has been shown to reduce strokes and systemic embolism as well as improve survival in AF/AFL patients [124,125], its underuse is a worldwide problem [41,126], which is even more pronounced in many regions of SSA [18,20,38,44]. Permanent and persistent AF are more common in SSA while paroxysmal AF is most frequent in HIC of the Western World, suggesting that patients are presenting late in the natural history of the possible underlying AF-causing cardiovascular diseases in SSA [41].

This systematic review has shown that about four in five of all AF are managed via rate-control strategy. These findings are similar to those of one other AF review in this region [122]. This demonstrates that evidenced-based AF rhythm-control strategies with catheter ablations which have been shown to have a survival benefit in AF patients with heart failure as in the CASTLE AF trial and others in a systematic review [127] are unlikely to become routine practice soon in this region. The same goes for VT ablations which are associated with significant morbidity, though not mortality, benefit [128].

### Supraventricular tachycardias

Adult studies on SVTs in SSA remain largely non-existent except for one surgical case series on Wolff-Parkinson-White syndrome, with no palpable epidemiological data on AVNRT, pre-excitation syndromes with their associated orthodromic and antidromic AVRT, atrial tachycardia, inappropriate sinus tachycardia, and postural orthostatic tachycardia syndrome (POTS). The lack of electrophysiological expertise in almost all SSA countries except in South Africa, underscores this non-existent literature of SVTs. In Western World clinical studies, AVNRT accounts for about 55–60% of all SVTs, AVRT 25–30%, and AT 10–17% [123,129], which is similar to what was seen in the only study in SSA. Most SVTs are very responsive to beta-blockers and non-dihydropyridine calcium channel blockers which are generically cheap in SSA and will be useful once the diagnosis is confirmed. SVTs can lead to tachycardia-induced cardiomyopathy, atrial fibrillation, and SCD especially if people with pre-excitation develop AF/AFL [123,130], and it is not unreasonable to speculate this as the etiology of some of the SCD seen in SSA. Emphasis on teaching, recognition, diagnosis, management, clinical relevance, and awareness of these SVTs and other cardiac arrhythmias need to be enforced in medical schools and allied medical training programs across SSA.

### Sudden cardiac arrest/sudden cardiac death

Given sparsity of structured Emergency Medical Services (EMS) and registries, the epidemiology of SCA/SCD is less characterized in SSA, apart from few data without supporting background for establishing reliable incidence estimates and etiologies. Both the internal and external validity of study results are affected by ascertainment of SCA/SCD cases. Data are more likely to reflect reality in the HIC with well-structured and readily available EMS of within a few minutes from the cardiac arrest, compared to poorer regions of SSA where these services are only present in patches in some urban areas, but remain largely inexistent in rural areas. Thus reported incidences of OHCA in SSA of 6–34 cases per 100,000 inhabitants are lower than in Europe and North America, where incidences of EMS-assessed and EMS-treated OHCA are about 86–110 per 100,000 and 40–57 per 100,000 respectively, and the presence of shockable rhythm (VT/VF) is seen in 20–35% of OHCA cases in adults and about 7% in children. In these regions, the incidence of IHCA varies from 0.6–1.6 per 1000 inpatient bed-days (VT/VF 16.1%, PEA 52.3%, asystole 23.6%, unknown 8% in adults, while VT/VF was 10.7% in children) [123,131].

This review identified underlying etiologies of SCA/SCD and ventricular arrhythmias in SSA, with hypertensive heart disease, coronary disease, cardiomyopathy, including peripartum cardiomyopathy, and valvular heart disease, especially rheumatic heart disease being the most common causes of SCA/SCD among adults [61,64,67,69,71,72,73,90]. Others include malignant arrhythmogenic inherited diseases [74,75,76,77,78,79,80,81,84,85,86,87,88,102,103,104], pericarditis [92], endemic parasitic infections [9,93], pulmonary embolism [62,91], endomyocardial fibrosis [95], congenital heart disease [96], pulmonary embolism [62,91], pulmonary hypertension [61], and aortic dissection/rupture [64]. Despite sarcoidosis being prevalent in SSA where approximately one in five cases is often mis-diagnosed as tuberculosis, studies on cardiac manifestations of sarcoidosis are lacking in this region [102,103,104]. However, it is known that African Americans in USA have a higher sarcoid incidence and >10 folds higher mortality including SCD and heart failure death compared to Caucasians [132]. Also, 5% of patients with sarcoidosis are known to have clinically manifest cardiac involvement and another 20–25% have asymptomatic cardiac involvement, and these manifestations include cardiomyopathy leading to heart failure, VT/VF, and cardiac conduction disease [133]. In HIC, the most frequent cause of OHCA is CAD accounting for more than 50% of SCA/SCD cases [123], with autopsy studies showing 80% of adults who suffer SCD have severe CAD [134], and in 61% of OHCA, at least one significant coronary lesion deemed responsible for the OHCA was seen on angiography in adults [135]. Autopsy studies of SCD also show that 10–15% have dilated or hypertrophic cardiomyopathy, and 5–10% have structurally normal hearts. About 30–50% of heart failure patients will die from SCD [134].

### Cardiopulmonary resuscitation

This review identified very low CPR awareness even among physicians and low rates of CPR initiation in SSA. Compare this to HIC of Western Europe and North America where 40–45% of OHCA victims received bystander CPR, and where >60% of the general population are trained to perform CPR, and where all IHCA victims without prior ‘do not resuscitate’ (DNR) code are expected to have full attempt at resuscitation via CPR and advanced cardiac life support [123]. The critical importance of quality CPR on survival in SCA victims have been demonstrated [136], and 12.6% with versus 7.6% without bystander CPR survive to discharge in large Western World registries [123]. For every minute that passes between collapse and defibrillation, survival from witnessed VF SCA falls 7% to 10% if no CPR is provided and when bystander CPR is provided, the fall in survival is more gradual and averages 3% to 4% [137]. Pre-emptive strides to inculcate at least chest-compression and rescue breathing CPR to the masses, as well as setting-up EMS and maintaining acceptable standards, alongside building new or improving existing recipient hospitals in terms of cardiac professional expertise and cardiac equipment, should become priority in eyes of policy makers and stakeholders within the health sector in SSA.

### Bradyarrythmias and cardiac implantable electronic devices (CIEDs)

It is estimated that 1 million patients worldwide die annually because of a lack of bradyarrhythmia device therapy [138], and with low implant rates in SSA, it is less doubtful that this region is contributing abundantly to this death pool. The rates of CIEDs implants in SSA are abysmally rock-bottom. For instance, Nigeria offers 0.2 implants per million population, which is >4000 times less than in Germany [12,14,139]. This means that many patients in this region with advanced bradyarrhythmias requiring pacemaker implantation are at the mercy of either succumbing to unbidden recurrent presyncope/syncope or premature sudden cardiac death. More than half (52%) of the patients identified as having an indication for pacing by visiting humanitarian pacing missions across 14 countries in SSA died before the missions arrived [110]. CAD and age-related degenerative conduction disease are the most common causes of AV block and SSS in HIC. The relatively low rates of CAD in SSA coupled with deaths from competitive causes at relatively younger age in SSA, could account for some of the low rates of pacemaker implantations in SSA. However, the approximately more than 200-fold lower rate of cardiac device implants compared to HIC of Western Europe and North America might not be fully explained by these alone [11,12]. That said, data from a few observational studies in the USA suggest lower risk of sick sinus syndrome in African Americans compared to Caucasians [140].

### Post-mortem or reusable CIEDs

Three identified main barriers to pacemaker and ICD implantation in SSA are reduced availability of implanting facilities with appropriate equipment, deficits in trained clinical specialists, and high cost of the devices and their accessories in the setting of high pay-out-of-pocket policies [9,12,106,107,141]. An interim solution to the high cost of cardiac devices is the re-use of previously implanted and explanted devices donated from the developed world, the so-called postmortem pacemakers and defibrillators, which have been shown to be safe in SSA [107,116,141,142] and worldwide [138,143], and their use has been backed by electrophysiology specialists [144]. Despite earlier observation of underuse of these recycled cardiac devices in SSA [12], growing partnerships between PASCAR and My Heart Your Heart (University of Michigan, USA) as well as Pace 4 Life (UK-based charity organisation) are now helping to bring more of these reconditioned CIEDs to SSA [9,141]. Given the palpable clear life-saving contribution of CIEDS, fostering partnerships and encouraging the re-use of CIEDS donated from the developed countries, plus initiatives aimed at building acceptable implanting centers and training specialists even through short and tailored fellowships [106], are of paramount importance and urgency.

### Possible reasons for under-diagnosis and undertreatment of arrhythmias in SSA

#### Insufficient and skewed budget allocations

Encouraging member States of the African Region of the World Health Organization to meet the prescribed target of 15% of annual expenditure on health under the Abuja Declaration, as majority are still falling short, will help [145]. Also, balancing the currently skewed budget allocations appropriately between communicable disease and NCDs will be helpful [145,146].

#### Insufficient health infrastructure including arrhythmia services

This review has observed deficiencies in health care systems and specialist cardiac services to manage CVDs [11,12,147,148]. Therefore, patients who survive OHCA, for example, and reach hospitals in SSA have lower chances of survival compared to their high-income country counterparts where invasive investigations and treatments are now routine practice. Every country in SSA should strive to have at least one large tertiary referral academic center for treatment of CVDs and invasive treatment of arrhythmias. The development of cardiac arrhythmia services with available ECG machines, built-in cardiac rhythm monitoring systems and devices like Holter monitors, external loop and patch recorders, mobile cardiac telemetry (MCT), and implantable loop recorders, as well as trained professionals to interpret their findings are warranted in order to diagnose arrhythmias in SSA.

#### Scarcity of cardiac professionals including electrophysiologists

There is a very low proportion of physicians to population, with majority of SSA countries having <5 physicians per 10,000 people [149], and 18% of the sub-Saharan African countries in a survey did not have a registered cardiologist, let alone a cardiac electrophysiologist [11,12]. The paucity of good training programs for cardiologists in SSA is compounded by the difficulty of African-trained physicians to get into good cardiology training fellowship programs in the western world, as cardiology is a highly attractive sub-specialty for which entry is usually fairly competitive even for western-trained physicians within their own respective countries. International and regional training partnerships should be fostered, like the PASCAR Fellowship in cardiac pacing which has already trained some fellows from countries where no pacing was present, and other regional initiatives [150].

#### Hight cost of arrhythmia management compounded by rarity of health insurance systems

Management of CVDs can very expensive [123]. CIEDs are very costly and unfordable by majority of the population in SSA, where direct out-of-pocket payments as a share of total health expenditure are still >40%, often leading to impoverishment [146]. This is compounded by the rarity of national health insurance systems, available in only about 15% of 55 African countries [149]. Development of these insurance systems should be encouraged and should become salient schemes in public health and financial planning within countries. An inclusive universal healthcare system with national-level health insurance scheme is probably better as it will avoid the poorer population from being left behind.

#### Inadequate epidemiological data

This review has noted very sparse data on SVTs, ventricular arrhythmias, and bradyarrhythmias. Poor ascertainment and capture of the true burden and trends of arrythmias and other CVDs might lead to underestimated disease rates and distort public health planning. Efforts should be made by governments and academic institutions through funding to remedy this handicap.

## Limitations

As recommended in PRISMA-P guidelines, classical publication databases such as PubMed/MEDLINE and EMBASE were used to retrieve the information. The databases used were supplemented by a database focused on African publication (AJOL). Furthermore, in order to capture any grey publication, some manual searches were conducted on internet and bibliography of published articles. Although, this wide variety of sources provide an accurate picture of the cardiac arrhythmias in sub-Saharan Africa, it comes with several challenges. Firstly, the clinical heterogeneity materialized, for instance, by marked differences observed in how studies were designed, differences in participants (age), comorbidities assessed, and treatment availability (Tables 1, 2, and 4). Secondly, the statistical heterogeneity characterized by how findings were reported precluded a meta-analysis. Although search criteria which combine the names of all sub-Saharan African countries was an option, we believe relevant arrhythmia studies in this region were not missed with the criteria used.

## Conclusions

The SSA region appears unprepared for the growing burden of arrhythmias, which appear to be under-diagnosed and undertreated. While victims of OHCA arrest in this region have low chances of ROSC and survival due to lack of CPR awareness and shortage of EMS, survivors to hospital also have lower survival rates due to sparsity of invasive cardiac procedures like coronary angiography, primary PCI, pacemakers, defibrillators, and antiarrhythmic medications. On the other hand, the majority of the tachyarrhythmias are managed conservatively due to low rates of invasive cardiac electrophysiological procedures in SSA, as setting-up health systems for their management is usually very expensive. Thus, to reduce morbidity and mortality from arrhythmias, high level strategic planning is needed, involving governmental, non-governmental organizations, international organizations, societies and associations, and local stakeholders.

## Abbreviations

AF/AFL: Atrial fibrillation/atrial flutter

CIEDs: Cardiac implantable electronic devices

CPR: Cardiopulmonary resuscitation

CRT: Cardiac resynchronization therapy

CVDs: Cardiovascular diseases

HIC: High-Income Countries

GBD: Global burden of disease

ICD: Implantable cardioverter defibrillator

IHCA: In-hospital cardiac arrest

NCDs: Non-communicable disease

PEA: Pulseless electrical activity

PPM: Permanent pacemaker

OHCA: Out-of-hospital cardiac arrest

RHD: Rheumatic heart disease

ROSC: Return of spontaneous circulation

SVTs: Supraventricular tachycardias

SCA: Sudden cardiac arrest

SCD: Sudden cardiac death

SSA: Sub-Saharan Africa

VT: Ventricular tachycardia

VF: Ventricular fibrillation
